# The Transition of Cardiovascular Disease Risks from NAFLD to MAFLD

**DOI:** 10.31083/j.rcm2406157

**Published:** 2023-05-31

**Authors:** Zifeng Yang, Juan Yang, Jingjing Cai, Xiao-Jing Zhang, Peng Zhang, Zhi-Gang She, Hongliang Li

**Affiliations:** ^1^Department of Cardiology, Renmin Hospital of Wuhan University, 430000 Wuhan, Hubei, China; ^2^Institute of Model Animal, Wuhan University, 430000 Wuhan, Hubei, China; ^3^Department of Cardiology, Huanggang Central hospital of Yangtze University, 438000 Huanggang, Hubei, China; ^4^Huanggang Institute of Translational Medicine, 438000 Huanggang, Hubei, China; ^5^Department of Cardiology, The Third Xiangya Hospital, Central South University, 410000 Changsha, Hunan, China; ^6^School of Basic Medical Sciences, Wuhan University, 430000 Wuhan, Hubei, China; ^7^Gannan Innovation and Translational Medicine Research Institute, Gannan Medical University, 341000 Ganzhou, Jiangxi, China

**Keywords:** nonalcoholic fatty liver disease, metabolic-associated fatty liver disease, cardiovascular disease risk

## Abstract

The increased burden of nonalcoholic fatty liver disease (NAFLD) parallels the increased incidence of overweight and metabolic syndrome worldwide. Because of the close relationship between metabolic disorders and fatty liver disease, a new term, metabolic-related fatty liver disease (MAFLD), was proposed by a group of experts to more precisely describe fatty liver disease resulting from metabolic disorders. According to the definitions, MAFLD and NAFLD populations have considerable discrepancies, but overlap does exist. This new definition has a nonnegligible impact on clinical practices, including diagnoses, interventions, and the risk of comorbidities. Emerging evidence has suggested that patients with MAFLD have more metabolic comorbidities and an increased risk of all-cause mortality, particularly cardiovascular mortality than patients with NAFLD. In this review, we systemically summarized and compared the risk and underlying mechanisms of cardiovascular disease (CVD) in patients with NAFLD or MAFLD.

## 1. Introduction

Nonalcoholic fatty liver disease (NAFLD) is a disease that is characterized by the accumulation of fat in the liver without excessive alcohol intake and other liver diseases [1, 2, 3]. NAFLD comprises a wide spectrum of liver diseases, ranging from simple steatosis to nonalcoholic steatohepatitis (NASH), advanced fibrosis, and cirrhosis [4]. During the past several decades, NAFLD has become one of the most prevalent chronic liver diseases and affects approximately 25% of the global population [5, 6].

Although NAFLD primarily manifests in the liver, it is a multisystemic disease affecting some extrahepatic organs [7]. As a result, NAFLD increases the risk of other diseases such as chronic kidney disease (CKD), type 2 diabetes mellitus (T2DM), and cardiovascular disease (CVD) [8, 9]. There is a strong relationship between NAFLD and CVD [10, 11, 12]. The potential mechanisms linking NAFLD and CVD, include insulin resistance, oxidative stress, chronic inflammation, hyperlipidemia, and endothelial dysfunction [13, 14, 15]. Moreover, an increasing number of studies have identified NAFLD as a risk factor for CVD [16, 17, 18].

Recently, based on the strong relationship between metabolic disorders and fatty liver disease, a new term, metabolic-associated fatty liver disease (MAFLD), has been introduced by a group of experts to more precisely describe fatty liver disease resulting from metabolic disorders [19, 20, 21]. MAFLD is defined as hepatic steatosis with any of the following conditions: overweight or obesity, presence of T2DM or metabolic disorders [22]. According to the criteria of NAFLD and MAFLD, nearly eighty percent of patients with liver steatosis can fulfill the criteria of NAFLD and MAFLD simultaneously [23, 24]. However, a number of patients who only meet one of the criteria still require consideration. For example, lean NAFLD individuals without systemic metabolic disorders cannot be diagnosed with MAFLD, and MAFLD individuals with alcoholic liver disease or other chronic liver diseases cannot be diagnosed with NAFLD [25, 26]. Therefore, individuals with hepatic steatosis can be divided into three groups, individuals with both MAFLD and NAFLD (NAFLD-MAFLD), individuals with only NAFLD but not MAFLD (NAFLD-only), and individuals with only MAFLD but not NAFLD (MAFLD-only). The transition from NAFLD to MAFLD inevitably has a significant impact on clinical practices, including the diagnosis, intervention approach, and risk of comorbidities.

Here, we review the history of NAFLD, MAFLD, and the transition from NAFLD to MAFLD. We further compare the cardiovascular risk between the NAFLD population and the MAFLD population and detail the differences in CVD risk among the NAFLD-only, MAFLD-only, and NAFLD-MAFLD overlapped groups.

## 2. From NAFLD to MAFLD

With the accumulation of in-depth mechanistic studies regarding the development of NAFLD, various metabolic disorders have been considered as main drivers of the occurrence and progression of NAFLD [2, 27, 28]. However, the diagnosis of NAFLD is based on the presence of excessive fat accumulation in the liver and without excessive alcohol intake and other etiologies of chronic liver disease, but it does not consider underlying metabolic disorders [29]. Therefore, a novel nomenclature that focuses mainly on systematic metabolic disorders, MAFLD, has been proposed, and MAFLD is an inclusive diagnosis [20].

### 2.1 History from NAFLD to MAFLD

In 1845, Addison first describe the term fatty liver. In 1964, the pathological mechanism of intrahepatic fat accumulation was first proposed [30]. In 1980, Ludwig [31] found steatohepatitis in liver biopsies from 20 individuals without alcohol abuse and other liver-damaging factors and thus named it NASH. In 1986, Schaffner and Thaler [32] proposed the concept of NAFLD and suggested that NASH should be regarded as a serious subtype of NAFLD. It was not until 1995 that NAFLD was proposed as a risk factor for CVD, which promoted an upsurge in NAFLD studies in recent decades [33]. The first NAFLD guideline was published by American scholars in 2002 [34]. Other countries and regions have also greatly increased their research interest in NAFLD and then issued corresponding guidelines [35, 36, 37]. In recent decades, with the prevalence of overweight, T2DM and metabolic dysregulation, NAFLD has been a leading cause of advanced liver diseases worldwide [38, 39, 40].

Metabolic disorders play a vital role in NAFLD, and the exclusion diagnosis strategies of NAFLD face many challenges such as the heterogeneous clinical outcomes of NAFLD and the lack of a uniform standard for the accurate calculation of alcohol intake [4, 41, 42]. In 2019, Eslam, Sanyal & George *et al*. [43] proposed the need for a new definition for fatty liver diseases, which foreshadowed the emergence of a novel nomenclature MAFLD the following year. The international expert group unanimously recommended redefining fatty liver disease related to metabolic disorders [20]. The proposal included using a new disease nomenclature, MAFLD, to renovate its former name NAFLD. MAFLD is diagnosed based on hepatic steatosis, similar to the diagnosis of NAFLD, but the diagnosis of MAFLD is a positive diagnosis and MAFLD can be combined with alcoholic fatty liver disease (AFLD) or other chronic liver diseases, which are common in life. Furthermore, MAFLD emphasizes the relationship between metabolic disorders and fatty liver. Currently, this name change has been endorsed by the Latin American Association for the Study of the Liver, the Asia Pacific Association for the Study of the Liver, the Chinese Society of Hepatology, and the Arabic Association for the Study of Diabetes and Metabolism [44, 45, 46, 47]. Over 1000 individuals who represent various professional institutions and doctors also support the change of terminology [48]. However, thus far, the American Association for the Study of Liver Diseases has not approved this name change [49]. In addition, a group of hepatologists, considering the current awareness of diseases among nonhepatologists, drug development, and the discovery of biomarkers, openly opposed the change of definition to MAFLD [50]. In summary, the diagnosis of MAFLD is a positive diagnosis, which emphasizes the impact of metabolic dysfunction on patients. This name change is supported by many regions and stakeholders. However, some hepatologists have expressed concern that this is a premature change in terminology. Changing the name from NAFLD to MAFLD may cause nonhepatologists to be more confused about this disease. The change may also have a negative impact on research development such as drug development and biomarker discovery. Thus, it is not clear whether the change of definition to MAFLD promotes the development of this field or leads to some unnecessary confusion and regression. Therefore, it is necessary to carefully evaluate the impact of this name change on different aspects such as disease awareness, drug development, and biomarker discovery, to judge the appropriateness of the renaming.

### 2.2 Comparing the Criteria of NAFLD with MAFLD

The criteria of NAFLD and MAFLD are both based on liver steatosis, but the renaming from NAFLD to MAFLD has also brought some internal changes.

NAFLD is defined by (1) fat accumulation in the liver as determined by imaging or histology, and (2) without other causes of fatty liver disease, including excessive alcohol abuse, viral infection, and hereditary disorders [51, 52]. MAFLD is diagnosed based on imaging, histological, or blood biomarker evidence of fatty liver, and the presence of at least one of the following three conditions: overweight/obesity, the presence of diabetes mellitus, or lean/normal weight with evidence of metabolic disorders [53, 54]. Metabolic disorders were defined by the presence of at least two of the following metabolic risk abnormalities: (1) waist circumference ≥102 cm in Caucasian men and waist circumference ≥88 cm in Caucasian women (or ≥90/80 cm in Asian men and women); (2) systolic blood pressure ≥130 mmHg and diastolic blood pressure ≥85 mmHg or hypertension drug treatment; (3) plasma triglycerides ≥150 mg/dL or its drug treatment; (4) plasma high-density lipoprotein cholesterol (HDL-C) <40 mg/dL for men and <50 mg/dL for women or the usage of specific drug treatment; (5) diagnosis of prediabetes or homeostasis model assessment of insulin resistance (HOMA-IR) score ≥2.5; and (6) plasma high-sensitivity C-reactive protein (hsCRP) level >2 mg/L [53].

**Fig. 1.**
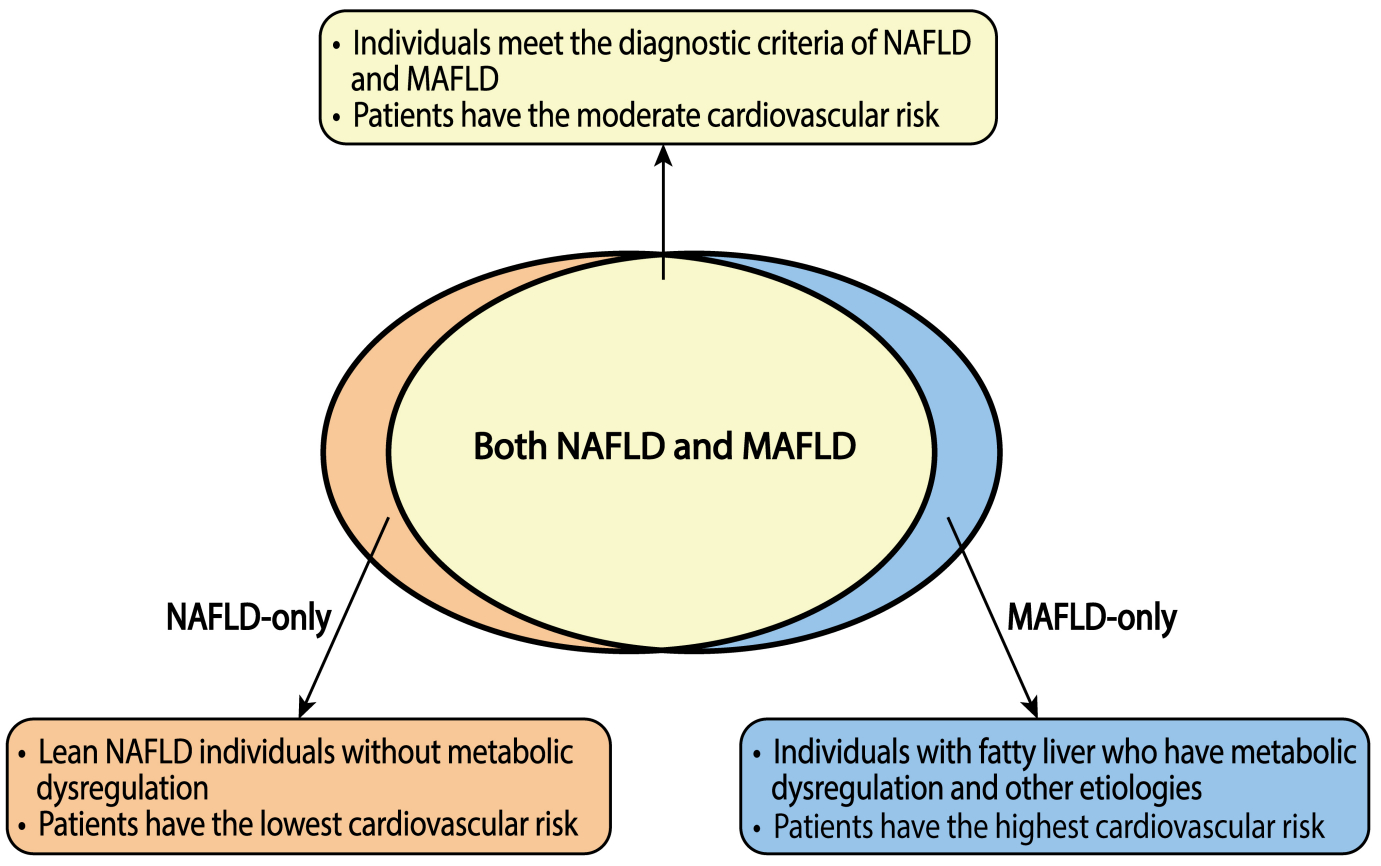
**Cardiovascular risk in the populations with NAFLD and MAFLD**. The brown area on the left represents the population that only meets NAFLD diagnostic criteria, named the NAFLD-only group; the yellow area in the middle represents the population that meets the diagnostic criteria of NAFLD and MAFLD, termed the NAFLD-MAFLD group; the blue area on the right represents the population only meets the MAFLD diagnostic criteria, called the MAFLD-only group. The cardiovascular risk is the highest in the MAFLD-only group, followed by the NAFLD-MAFLD and the NAFLD-only groups. NAFLD, nonalcoholic fatty liver disease; MAFLD, metabolic-associated fatty liver disease.

The diagnostic criteria of NAFLD and MAFLD both include pathological liver steatosis and imaging features of fatty liver. In addition, the diagnosis of MAFLD can also be based on blood biomarker evidence of fatty liver. Most individuals with hepatic steatosis fulfill the diagnostic criteria of NAFLD and MAFLD [55]. There are also differences in the diagnostic criteria of NAFLD and MAFLD. NAFLD is a negative, exclusion criterion that needs to exclude liver diseases caused by alcohol and other reasons. In contrast, MAFLD is a positive, inclusion criterion that emphasizes the role of obesity, diabetes, and metabolic disorders in fatty liver, which can combine with other chronic liver diseases. The classification of individuals with hepatic steatosis can be redefined through these two different diagnostic criteria. About 80% of patients meet the diagnostic criteria of NAFLD and MAFLD, which can be classified as both NAFLD and MAFLD (NAFLD-MAFLD) [25]. About 15% of patients fulfill the criteria for MAFLD but not NAFLD, which can be classified as the MAFLD-only group [56]. This group includes individuals with hepatic steatosis who have metabolic dysregulation and other etiologies, including alcohol and viral infection [57]. In addition, about 5% of patients fulfill the criteria for NAFLD but not MAFLD, which can be classified as the NAFLD-only group [56]. This group includes lean NAFLD individuals without metabolic disorders [58]. These groups are described in Fig. 1.

### 2.3 Advantages and Disadvantages of the Conversion from NAFLD to MAFLD 

Renaming from NAFLD to the new term MAFLD brings some advantages and disadvantages. These advantages and disadvantages can be described in terms of diagnosis, treatment, and prevention.

First, from the perspective of diagnosis, MAFLD criteria can better help identify patients with long-term hepatic and extrahepatic adverse consequences than the diagnostic criteria of NAFLD [59, 60, 61]. This means that patients with high risks of developing serious liver outcomes and complications can be widely screened. In addition, the new term MAFLD attaches importance to the role of overweight, metabolic disorders, and T2DM in fatty liver disease, which can enhance the awareness of fatty liver disease and the ability to diagnose fatty liver disease in the clinic [62, 63]. Second, the diagnosis of NAFLD needs to exclude other liver diseases while MAFLD can combine with other secondary liver diseases. Thus, the definition of MAFLD allows us to consider other liver diseases that may accompany NAFLD and patients can be treated more widely. Third, the term MAFLD includes “metabolic”, which may increase public awareness of the tight relationship between fatty liver and metabolism. Thus, more public attention would be given to metabolic health to prevent fatty liver.

However, the change in terminology also brings some potential disadvantages. First, the definitions of NAFLD and MAFLD are slightly different, so the individuals did not completely overlap. For example, individuals with lean NAFLD may be overlooked by MAFLD [58]. In addition, a majority of noninvasive biomarkers and scores are derived using patients with NAFLD/NASH, rather than in patients with MAFLD [64, 65]. For example, a NIS4 biomarker panel was developed using NASH patients, which leaves uncertainties in the accuracy of identifying hepatitis in patients with MAFLD [66]. Second, although there are no drugs approved by the FDA for NASH at present, some drugs, such as elafibranor, and obeticholic acid, showed encouraging results in the treatment of NASH in phase 2 or 3 clinical trials [67, 68]. MAFLD can coexist with other liver diseases. Thus, the heterogeneity of patients is higher and the efficacy of the testing reagents in ongoing clinical trials is impacted.

## 3. NAFLD/MAFLD is a Risk Factor for CVD

**Fig. 2.**
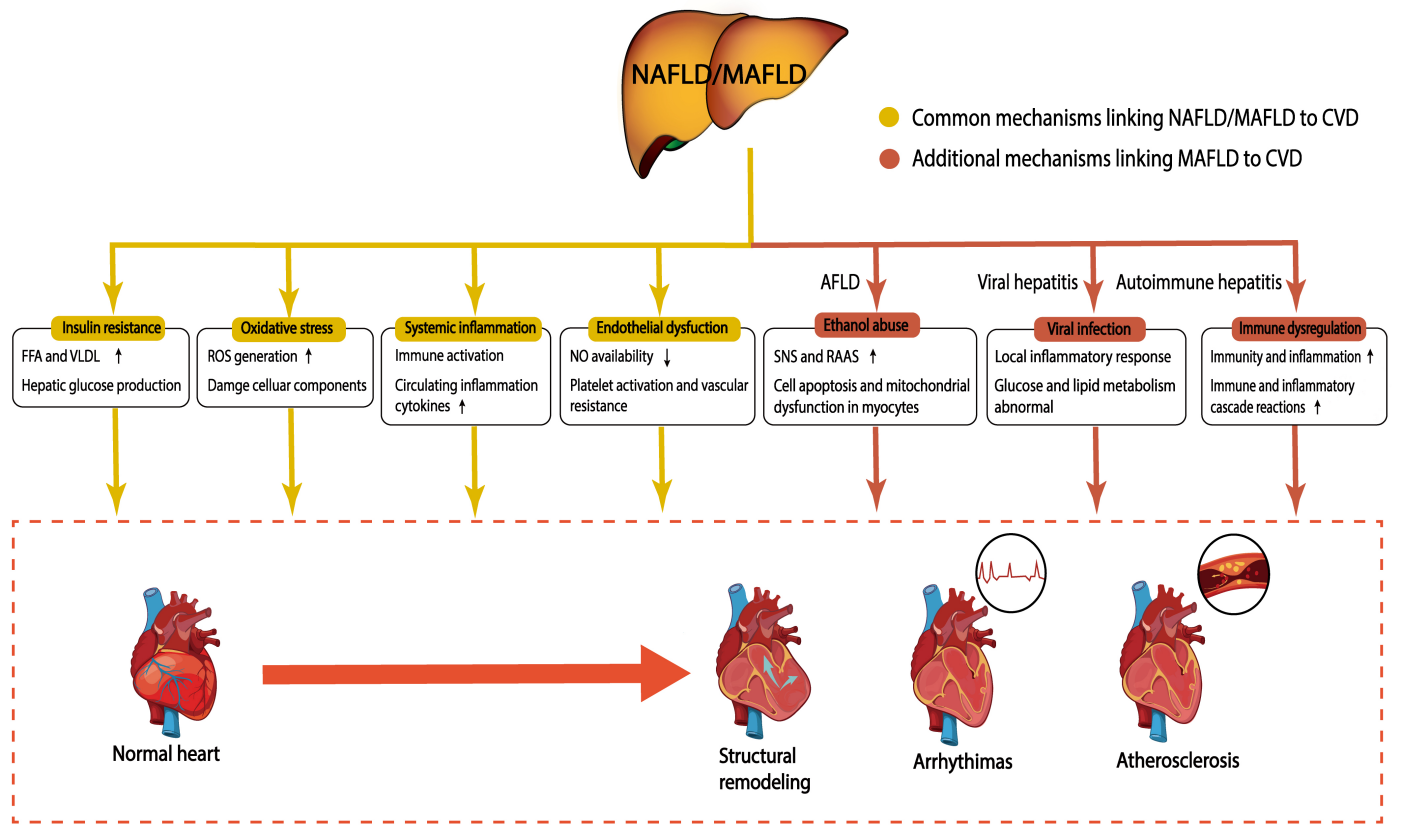
**Potential mechanisms linking NAFLD/MAFLD to CVD**. NAFLD/MAFLD promotes CVD through potential pathophysiological mechanisms, including insulin resistance, oxidative stress, systemic inflammation, and endothelial dysfunction. In addition to these mechanisms, patients with MAFLD may company with impairments from ethanol, viral infection, and immune dysregulation, which further increase the risk of CVD. The yellow boxes represent common mechanisms linking NAFLD/MAFLD to CVD; the red boxes represent additional mechanisms linking MAFLD to CVD. NAFLD, nonalcoholic fatty liver disease; MAFLD, metabolic-associated fatty liver disease; CVD, Cardiovascular disease; FFA, free fatty acid; VLDL, very low-density lipoprotein; ROS, reactive oxygen species; NO, nitric oxide; AFLD, alcoholic fatty liver disease; SNS, sympathetic nervous system; RAAS, renin-angiotensin-aldosterone system.

Although a causal relationship between NAFLD and CVD has not been determined, potential mechanisms linking NAFLD to CVD have been explored for over a decade [69, 70, 71]. MAFLD has been recognized as a fatty liver disease resulting from metabolic disorders. Patients who are diagnosed with MAFLD have at least two metabolic disorders or other liver diseases. Thus, individuals with MAFLD may have a higher cardiovascular risk than individuals with NAFLD (Fig. 2).

### 3.1 Potential Mechanisms Linking NAFLD to CVD

It has been indicated that NAFLD can promote the development of CVD independent of traditional CVD risks. Some potential pathophysiological mechanisms linking NAFLD to CVD comprise insulin resistance, oxidative stress, systemic inflammation, and endothelial dysfunction [72].

Insulin resistance is an important feature of NAFLD and plays a crucial role in CVD pathogenesis [73, 74]. Insulin resistance would cause hyperglycemia by reducing glucose uptake and can lead to the export of peripheral free fatty acids (FFAs) to the liver [75, 76]. More importantly, insulin resistance would contribute to an elevated level of insulin. Increased insulin further induces lipid accumulation in the liver through accelerating glycogenesis and de novo lipogenesis [77, 78]. At the same time, increased lipid accumulation in the liver can further deteriorate insulin resistance in individuals with NAFLD [79]. Eventually, these constitute a vicious circle, leading to the increasing accumulation of fat in the liver. Persistent hyperglycemia and insulin resistance activate inflammation and lead to abnormal lipoprotein metabolism, which induces the occurrence of atherosclerotic cardiovascular disease (ASCVD) [80]. Insulin clearance further worsens this situation in patients with NAFLD. In addition, hyperinsulinemia alters the activities of lipogenic enzymes and leads to the mobilization of subcutaneous fat to deposit in viscera [81]. Mobilized fat also increases very low-density lipoprotein (VLDL) levels and circulating FFAs, which contribute to atherosclerosis [82, 83]. Moreover, atherosclerotic dyslipidemia is also attributed to increased very low-density lipoprotein synthesis and decreased FFA oxidation and triglyceride (TG) output in individuals with NAFLD [84, 85].

Oxidative stress is also a critical mechanism linking NAFLD to CVD [86]. Excessive fat accumulates in hepatocytes inducing reactive oxygen species (ROS) overproduction in the mitochondria and endoplasmic reticulum [75]. Excessive ROS overflow into the circulation and increase circulating levels of oxidative stress markers, such as serum soluble NOX2-derived peptide (sNOX2-dp) and urinary 8-iso-prostaglandin F2α (8-iso-PGF2α), in NAFLD [87, 88]. Some studies indicate that the levels of urinary 8-iso-PGF2α and serum sNOX2-dp increase with the severity of hepatic steatosis in NAFLD patients [88]. It has been reported that urinary 8-iso-PGF2α is also an independent predictor of NAFLD [88]. Therefore, strong relationships between oxidative stress markers and NAFLD indicate that oxidative stress plays a vital role in the pathophysiology of NAFLD. Excess ROS in the circulation also damage cellular components of vascular cells, such as mitochondrial DNA and cell membrane, leading to endothelial dysfunction and atherosclerosis [16, 89, 90, 91]. Moreover, it has been reported that increased circulating levels of NOX2 and 8-iso-PGF2α are also closely associated with some CVDs such as coronary heart disease, atherosclerosis, and hypertension [92, 93].

At the same time, systemic inflammation also plays a critical role in linking NAFLD and CVD [86]. NAFLD leads to elevated levels of inflammatory mediators, such as intercellular adhesion molecule-1, P-selectin, interleukin-6, and hsCRP [94]. Increased levels of inflammatory factors would contribute to systemic inflammation, which poses a threat to the cardiovascular system [95]. Furthermore, the epicardial fat volume in patients with NAFLD is increased, which may increase the secretion of proinflammatory factors such as tumor necrosis factor-α, leptin, and interleukin 1-β [96]. These would affect the myocardium in a state of systemic inflammation [97].

Endothelial dysfunction is initiated from the early stage of atherosclerosis and is characterized by the decreased availability of nitric oxide (NO) [98]. Elevated levels of asymmetric dimethyl arginine (ADMA), an endogenous antagonist representing nitric oxide synthase, are prevalent in patients with NAFLD [99, 100]. The increase in ADMA levels results in a decrease in NO availability and endothelial dysfunction. Furthermore, the level of homocysteine is also elevated in patients with NAFLD [101]. Hyperhomocysteinemia causes oxidative stress by reducing the storage of glutathione, which is also related to a low level of NO, increased platelet activity, and vascular resistance [73].

### 3.2 Potential Mechanisms Linking MAFLD to CVD

Individuals with MAFLD have a higher burden from metabolic disturbances than individuals with NAFLD due to the diagnostic criteria. Thus, metabolic stress-triggered insulin resistance, oxidative stress, systemic inflammation, dyslipidemia, and endothelial dysfunction could be more conspicuous in MAFLD individuals. Additionally, the diagnosis of MAFLD does not exclude other liver diseases including AFLD, viral hepatitis, and autoimmune hepatitis. Therefore, potential mechanisms linking MAFLD to CVD are also affected by other factors, such as ethanol, viral infection, and immune dysregulation.

The cardiovascular system can be indirectly affected by chronic ethanol consumption. Chronic ethanol abuse increases the activity of the sympathetic nervous system (SNS) and the activity of the renin-angiotensin-aldosterone system (RAAS) [102]. The SNS and RAAS activation causes hypertension which increases the load on the heart and exacerbates alcoholic cardiomyopathy [102, 103]. In addition, its metabolite acetaldehyde can act as a direct toxin to cardiomyocytes. These effects can lead to cell apoptosis and mitochondrial dysfunction in myocytes, which will aggravate contractile dysfunction [104].

Viral hepatitis, such as that due to hepatitis C virus (HCV), directly or indirectly interferes with glucose and lipid metabolism, resulting in insulin resistance, steatosis, and T2DM [105, 106, 107, 108, 109, 110]. Furthermore, HCV in blood vessels directly causes a local inflammatory response, leading to the occurrence of CVD [111, 112].

Immune dysregulation in autoimmune hepatitis may also increase the risk of CVD. The enhancement of immune and inflammatory cascade reactions is related to endothelial dysfunction and ROS production [113, 114, 115]. In addition, immunity and inflammation themselves can mediate the occurrence and development of CVD [116, 117].

## 4. Individuals with MAFLD may be at a Higher Risk for CVD than Individuals with NAFLD

The change of definition to MAFLD is not only a change in nomenclature, but it also brings other effects, such as the different cardiovascular risks between patients with NAFLD and patients with MAFLD. Two aspects can reflect that patients with NAFLD and patients with MAFLD have different cardiovascular risks. On one hand, the cardiovascular risk is different between the NAFLD population and the MAFLD population. On the other hand, the cardiovascular risk is different among the MAFLD-only, NAFLD-MAFLD overlapped, and NAFLD-only groups. These are summarized in Table 1 (Ref. [60, 61, 118, 119, 120, 121, 122, 123, 124, 125, 126, 127, 128]).

**Table 1.** **Summary of clinical studies and meta-analysis on comparing CVD risk between NAFLD and MAFLD**.

Region	Study design	Fatty liver diagnosis	Study population	NAFLD and MAFLD	Main results	References
Comparison of cardiovascular risk differences between the NAFLD population and the MAFLD population						
Japan	Cross-sectional	Ultrasonography	765	541 NAFLD	The MAFLDs have higher BMI, LDL-c, TG, lower HDL-c, and higher risks for diabetes and hypertension than the NAFLDs.	[61]
				609 MAFLD		
Japan	Cross-sectional	Ultrasonography	2306 subjects with fatty liver	1462 NAFLD	MAFLD better helps identify patients with ASCVD risk than NAFLD.	[118]
				1859 MAFLD		
Japan	Cross-sectional	Ultrasonography	890 subjects who underwent health checkups	268 NAFLD	The MAFLDs have a higher risk of subclinical atherosclerosis than the NAFLDs.	[119]
				384 MAFLD		
Korea	Cross-sectional	Fatty liver index	9,584,399	2,680,217 NAFLD	The MAFLDs have a higher risk for CVD mortality than the non-MAFLDs (HR 1.46, 95% CI: 1.41–1.52); The NAFLDs have a higher risk for CVD mortality than the non-NAFLDs (HR 1.12, 95% CI: 0.96–1.30).	[60]
				3,573,644 MAFLD		
The United States	Cross-sectional	Ultrasound-fatty liver index	19,617 adults	6658 NAFLD	The MAFLDs and the NAFLDs have similar risks for CVD and CKD.	[120]
				7131 MAFLD		
The United States	Retrospective cohort	Ultrasonography	13,083	4347 NAFLD	The MAFLDs have higher BMI, HOMA-IR, lipids, and higher risks for diabetes and hypertension than the NAFLDs.	[121]
				3885 MAFLD		
The United States	Retrospective cohort	Ultrasonography	12,480	3909 NAFLD	The MAFLDs (HR 2.01, 95% CI: 1.66–2.64) have a higher risk for CVD-related mortality than the NAFLDs (HR 1.53, 95% CI: 1.26–1.86).	[122]
				3779 MAFLD		
Korea	Retrospective cohort	Ultrasonography	2144 subjects without a history of ASCVD	995 NAFLD	MAFLD criteria are better than NAFLD criteria in predicting ASCVD risk in asymptomatic subjects.	[123]
				891 MAFLD		
Sri Lankan	Prospective cohort	Ultrasonography	2985	940 NAFLD	The MAFLDs and the NAFLDs have similar new-onset metabolic traits and risks for CVD events.	[124]
				990 MAFLD		
China	Prospective cohort	Ultrasonography	6873	2771 NAFLD	The MAFLDs and the NAFLDs have similar risks for diabetes, CKD, and CVD.	[125]
				3212 MAFLD		
Europe, Asia, and North America	Meta-analysis	Imaging or biopsy	22 studies, 379,801 participants	Of 67,742 patients, 23,865 NAFLD. Whereas of 379,801 patients, 116,806 MAFLD	The MAFLDs have higher BMI, triglycerides, lower HDL-c, and higher risks for hypertension and diabetes than the NAFLDs.	[126]
Comparison of cardiovascular risk among the NAFLD-only, MAFLD-only, and NAFLD-MAFLD groups						
Korea	Cross-sectional	Fatty liver index	9,584,399	52,747 NAFLD-only	Compared to individuals without fatty liver disease, the risk for CVD events increased 2.33 (2.30–2.36) fold in the MAFLD-only group, 2.15 (2.13–2.17) fold in the NAFLD-MAFLD group, and 1.68 (1.59–1.78) fold in the NAFLD-only group. The MAFLD-only has the highest association with CVD-related death.	[60]
				870,818 MAFLD-only		
				2,625,321 NAFLD-MAFLD		
Japan	Cross-sectional	Ultrasonography	2306 subjects with fatty liver	301 NAFLD-only	The NAFLD-only have a lower incidence of CVD event than the NAFLD-MAFLD, with HR 0.70 (95% CI 0.50–0.98). The MAFLD-only has a similar risk of CVD events with the NAFLD-MAFLD, with HR 1.19 (0.89–1.58).	[118]
				698 MAFLD-only		
				1161 NAFLD-MAFLD		
The United States	Retrospective cohort	Ultrasonography	12,480	528 NAFLD-only	The risks for CVD-related mortality are different in the NAFLD-only, NAFLD-MAFLD, and MAFLD-only groups, with HRs 0.46 (0.20–1.02), 1.86 (1.51–2.28), and 2.35 (1.60–3.45), respectively.	[122]
				658 MAFLD-only		
				3251 NAFLD-MAFLD		
The United States	Retrospective cohort	Ultrasonography	13,640 adults aged ≥20 years	254 NAFLD-only	The MAFLD-only and NAFLD-MAFLD have more CVD risk factors than NAFLD-only. They also have a higher risk for CVD mortality than the NAFLD-only group, with HRs 9.4 (2.6–34.6) and 7.0 (2.1–23.1), respectively.	[127]
				503 MAFLD-only		
				2240 NAFLD-MAFLD		
The United States	Retrospective cohort	Ultrasonography	7761 participants	394 NAFLD-only	The MAFLD-only and the NAFLD-MAFLD groups have increased CVD risk factors compared to the NAFLD group. Compared to individuals without hepatic steatosis, the risks for CVD mortality were 2.59 (1.10–6.09), 1.95 (1.55–2.45), and 0.29 (0.10–0.86) in the MAFLD-only, NAFLD-MAFLD, and NAFLD-only groups, respectively.	[128]
				212 MAFLD-only		
				2044 NAFLD-MAFLD		

CVD, Cardiovascular disease; NAFLD, nonalcoholic fatty liver disease; MAFLD, metabolic-associated fatty liver disease; BMI, body mass index; HDL-c, high-density lipoprotein cholesterol; LDL-c, low-density lipoprotein cholesterol; TG, triglyceride; HOMA-IR, homeostasis model assessment-insulin resistance; ASCVD, atherosclerotic cardiovascular disease; HR, hazard ratio; CI, confidence interval; CKD, chronic kidney disease.

### 4.1 Comparison of Cardiovascular Risk Differences between the NAFLD Population and the MAFLD Population

Emerging evidence from population studies has indicated that individuals with MAFLD have a higher risk for development of other traditional CVD risk factors, CVD events, and CVD death than individuals with NAFLD [61, 121, 126]. In an observational data meta-analysis involving 379,801 participants, the association between MAFLD and NAFLD in cardiovascular disease risk factors was reported [126]. MAFLD was more relevant to hypertension, diabetes, high body mass index (BMI), and high lipid levels than NAFLD. Furthermore, other studies have also reported that patients with MAFLD have higher BMI, HOMA-IR, lipid levels, and a higher possibility of having diabetes and hypertension than patients with NAFLD [61, 121].

In addition, patients with MAFLD or NAFLD also have different risks for cardiovascular events. In a single-center and cross-sectional study, 2306 subjects with fatty liver were enrolled, and ASCVD risk was estimated by noninvasive tests such as the Suita score [118]. This report indicated that MAFLD is related to worsening of the Suita score and that MAFLD criteria better help identify patients with ASCVD risk than NAFLD criteria. A cross-sectional study also showed that individuals with MAFLD have a higher probability of coronary artery calcification than individuals with NAFLD, which is one of the markers of atherosclerosis [119]. Moreover, in a cross-sectional study, 2144 individuals who had no history of ASCVD were offered a health examination at a health center [123]. ASCVD risks can be identified by MAFLD and NAFLD criteria, but MAFLD criteria can better predict the risk of ASCVD than NAFLD criteria in asymptomatic subjects. This means that individuals diagnosed with MAFLD need to further enhance their awareness of ASCVD prevention, regardless of whether they have a history of ASCVD. Whether in NAFLD or MAFLD, the risk of CVD in patients with liver fibrosis is higher than that in patients with simple hepatic steatosis [129, 130]. Moreover, MAFLD criteria can better identify patients with advanced liver fibrosis than NAFLD criteria [61]. Similar to NAFLD, liver fibrosis can also increase the risk of cardiovascular events in MAFLD [131, 132]. In a retrospective study, the risk of cardiovascular events was compared between NAFLD and MAFLD populations and among MAFLD individuals with various degrees of liver fibrosis [132]. Compared with NAFLD individuals, MAFLD individuals have significantly higher 10-year CVD risks. More importantly, the risk of cardiovascular events is increased with the severity of liver fibrosis in MAFLD patients [132]. However, this study also has some shortcomings such as a small sample size. Therefore, larger clinical trials are needed to further study the impact of liver fibrosis on CVD risks in MAFLD individuals.

The differences in cardiovascular-related mortality are also striking between the NAFLD population and the MAFLD population. It was concluded by using data from the third National Health and Nutrition Examination Survey (NHANES III) that CVD-related mortality was slightly higher in the MAFLD population (hazard ratio (HR) 2.01, 95% CI: 1.66–2.64) than in the NAFLD population (HR 1.53, 95% CI: 1.26–1.86) [122]. In addition, the conclusion was also drawn from a nationwide health information database of the National Health Insurance Service in South Korea that the MAFLD population is significantly associated with CVD-related death (HR 1.46, 95% CI: 1.41–1.52) [60]. However, there is no relationship between NAFLD and CVD-related death (HR 1.12, 95% CI: 0.96–1.30). Therefore, the risks of cardiovascular events and cardiovascular mortality are higher in individuals with MAFLD than in individuals with NAFLD. This may be because metabolic disorders are closely related to CVD and have a synergistic effect with fatty liver on CVD. In addition, other liver diseases including AFLD and viral hepatitis can also increase the risk of CVD. Other studies have yielded different results that the NAFLD population and the MAFLD population had a comparable prevalence of nonfatal and fatal CVD events or similar clinical characteristics [120, 124, 125]. However, the prevalence of NAFLD is lower than that of MAFLD in their studies, which means that more individuals with MAFLD are at risk for CVD [120, 124, 125]. In summary, the MAFLD population is at a greater risk for CVD than the NAFLD population.

### 4.2 Comparison of Cardiovascular Risk among the NAFLD-Only, MAFLD-Only, and NAFLD-MAFLD Groups

According to the definition of NAFLD and MAFLD, individuals with hepatic steatosis can be roughly divided into three groups: the NAFLD-only, MAFLD-only, and NAFLD-MAFLD overlapped groups. In recent years, the risk of CVD among three groups has been reported. Emerging studies have indicated that the risk for developing other traditional CVD risk factors, CVD events, and CVD death is different among the NAFLD-only, MAFLD-only, and NAFLD-MAFLD overlapped groups.

In several studies, some traditional CVD risk factors, such as high levels of lipids and increased HOMA-IR, are more common in MAFLD-only and NAFLD-MAFLD overlapped groups than in the NAFLD-only group [122, 127, 128]. Other traditional CVD risk factors, such as overweight and diabetes, only appear in MAFLD-only or NAFLD-MAFLD overlapped groups [122, 127, 128]. However, there was no obvious difference in these traditional CVD risk factors between MAFLD-only and NAFLD-MAFLD overlapped groups. To sum up, the NAFLD-only group has the lowest risk for developing other traditional CVD risk factors while the MAFLD-only and NAFLD-MAFLD overlapped groups had a similar risk for developing other traditional CVD risk factors.

Furthermore, the risk of CVD events is also different among the three groups. In a nationwide cohort study, Lee *et al*. [60] collected data from 9,584,399 adults aged 40–64 years who were offered health examinations from 2009 to 2010. Of these participants, 354,886 individuals were classified into three groups. The MAFLD-only group and NAFLD-MAFLD overlapped groups have the highest cumulative incidence of CVD events, followed by the NAFLD-only group. After adjusting for complex factors, compared with individuals without fatty liver disease, the HR for CVD events was 2.33 (2.30–2.36) in the MAFLD-only group, 2.15 (2.13–2.17) in the NAFLD-MAFLD group, and 1.68 (1.59–1.78) in the NAFLD-only group. Tsutsumi *et al*. [118] recruited 2306 subjects with fatty liver, and the worsening of the ASCVD risk score was higher in the NAFLD-MAFLD overlapped and MAFLD-only groups than in the NAFLD-only group. However, there was no statistical significance in the risk of CVD events between MAFLD-only group and NAFLD-MAFLD overlapped group. In summary, the risk of CVD events was highest in the MAFLD-only and NAFLD-MAFLD overlapped groups while the NAFLD-only group has the lowest risk of CVD events.

In addition to the different risks for developing other traditional cardiovascular risk factors and cardiovascular events, cardiovascular mortality was also different among these three groups. In a population-based study, Kim *et al*. [128] collected data from 7761 participants from NHANES II, of which participants in the NAFLD-MAFLD overlapped group accounted for 23.5% of total participants, those in the MAFLD-only group accounted for 2.4%, and those in the NAFLD-only group accounted for 6.1%. In univariable Model 1, compared with individuals without hepatic steatosis, the HRs (95% CI) for cardiovascular mortality in the MAFLD-only, NAFLD-MAFLD overlapped, and NAFLD-only groups were 2.59 (1.10–6.09), 1.95 (1.55–2.45), and 0.29 (0.10–0.86), respectively. This indicates that the strongest relationship with CVD-related mortality was found for the MAFLD-only group, followed by the NAFLD-MAFLD overlapped group, and then by the NAFLD-only group. This is also reflected in some other studies. Huang *et al*. [122] also collected 12,480 participants aged 20–74 years in NHANES III; the HR (95% CI) for CVD-related mortality was 0.46 (0.20–1.02), 1.86 (1.51–2.28), and 2.35 (1.60–3.45) in the NAFLD-only, NAFLD-MAFLD and MAFLD-only groups, respectively. Similarly, Nguyen *et al*. [127] and Lee *et al*. [60] also reported that the highest cumulative incidence of CVD-related mortality was in the MAFLD-only group while the lowest cardiovascular mortality was in the NAFLD-only group.

As discussed above, the cardiovascular risk of individuals diagnosed by the criteria of MAFLD but excluded by the criteria of NAFLD may be the highest. The cardiovascular risk of individuals with NAFLD and MAFLD is intermediate, and the cardiovascular risk of individuals diagnosed by the criterion of NAFLD and excluded by the criterion of MAFLD is the lowest. This may be because the MAFLD-only population includes people who were previously excluded by NAFLD criteria such as individuals with viral hepatitis or alcoholic liver disease. Viral infections and alcohol intake are related to the development of traditional CVD risk factors and an increased risk of CVD [133, 134, 135]. These results also indicate that MAFLD criteria can better predict the high-risk population for CVD than NAFLD criteria. Individuals captured by MAFLD criteria need to increase their awareness of CVD prevention. Additionally, physicians should be vigilant and treat such patients as early as possible to reduce the risk of CVD.

## 5. Conclusions and Prospective

Since NAFLD was proposed as a CVD risk factor in 1995, NAFLD has received increasing attention. At present, several large population studies have suggested that NAFLD is an independent risk factor for CVD [16, 17, 18]. NAFLD and CVD share many traditional risk factors, in addition, NAFLD promotes the development of various CVDs independent of traditional risk factors. Considering the close relationship between metabolic disorders and NAFLD, a new terminology MAFLD has been proposed. Although the majority of patients diagnosed by NAFLD could be identified using MAFLD criteria, a small number of individuals are either diagnosed by NAFLD or MAFLD alone. On average, the MAFLD population may have a higher metabolic burden and risk for CVD than the population with NAFLD. The change in terminology has a strong influence on clinical practices regarding diagnosis, intervention, prevention, and the risk of comorbidities. However, whether this change results in an improvement in patient care remains to be studied in future trials.
